# Supplementary material for: Long-Term Outcome in Systemic Lupus Erythematosus; Knowledge from Population-Based Cohorts
Source: J Clin Med. 2021 Sep 22;10(19):4306. doi: 10.3390/jcm10194306 (PMC8509465; doi:10.3390/jcm10194306)
Supplement: Supplementary file 1 [file jcm-10-04306-s001.zip › jcm-1346641-supplementary.pdf]

Table S1: Case finding methodology and SLE ascertainment in population-based Systemic Lupus Erythematosus cohorts

Study location (ref.)	Inclusion period	Population at risk, n	Age, yrs	Case definition	Chart review	Control group	Sources of Retrieval/Case Ascertainment	Capture recapture
**Tucumán, Argentina [1]**	2005 - 2012	1 011 188	>16	≥ 4 ACR 82/97 criteria	Yes	Non	1. List of in and outpatients from four public hospital. 2. Rheumatologist in private practice	No
**Barbados [2]**	2000 - 2009	268 792	All	≥ 4 ACR 97 criteria	Yes	Non	1. List of all SLE patients from the only public and private rheumatologist service on the island. 2. All medical practitioners were contacted. 3. Patient organization	No
**Manitoba, Canada [3]**	1980 - 1996	1 100 295	All	≥ 4 ACR 82 criteria	Yes	Non	1. All general internist, rheumatologist, nephrologist and one oncologist in Manitoba. 2. 20 % of the region 500 + family physicians.	No
**Hong Kong, China* [4,5]**	1991 - 2006	~ 1 000 000	All	≥ 4 ACR 97 criteria	Yes	General population	List of in and outpatients of one regional hospital with no private practitioners in the region and no other public hospital	No
**Curaçao [6]**	1980 - 1990	146 500	All	≥ 4 ACR 82 criteria	Yes	Non	1. List of patients discharged from the only hospital at the island. 2. All specialists were ask to provide information of patients. 3. Death certificate from the Public Health Department	No
**Denmark [7,8]**	1975 - 1995	NA	All	≥ 4 ACR 82 criteria	Yes	General population; age and sex matched	List of in and out patients from eight hospital	No
**Funen, Denmark* [9,10]**	1980 - 2010	NA	≥15	≥ 4 ACR 82 criteria/ Clinical diagnosis	Yes	General population	1. A registry covering all in patients. 2. A registry covering all out patients. 3. General practitioners and private specialists .4. Hospital laboratory (ANA, anti dsDNA).	Yes
**Northwest Greece [11]**	1982 - 2001	488 435	All	≥ 4 ACR 82 criteria	Yes	General population	1. In and outpatients referred to rheumatology clinic from the two rheumatology clinics in the area. 2. Patients refereed to all private rheumatologist in the area.	No
**Crete, Greece [12]**	1999 - 2013	~ 650 000	> 15	≥ 4 ACR 97 criteria	Yes	Non	1. In and out patients from only rheumatology clinic and all nephrology, dermatology hospital departments in the region. 2. Private rheumatologists (9/12). 3. Laboratory, general practitioners, patients association, hospitalizations. 4. The National Renal Data System for ESRD	No

Study location (ref.)	Inclusion period	Population at risk, n	Age, yrs	Case definition	Chart review	Control group	Sources of Retrieval/Case Ascertainment	Capture recapture
**Iceland* [13,14]**	1957 - 2001	239 498	All	≥ 4 ACR 82 criteria	Yes	General population; life tables	1. Computerized search in all hospitals in Iceland. 2. Questionnaire sent to all private practitioners, district medical officers, internists, neurologists, pediatricians, dermatologists, nephrologists, rheumatologists	No
**Okinawa, Japan [15]**	1972 - 1991	NA	All	≥ 4 ACR 82 criteria	Yes	General population	1. Forty-five hospitals and seven clinics in the area, including all dialysis unit. 2. The Okinawa Medical Association. 3. A dialysis registry covering all chronic dialysis patients who survive more than 1 month	No
**Northern Norway* [16-18]**	1978 - 2006	224 724/226 898	≥16	≥ 4 ACR 82/97 criteria	Yes	General population; 5:1, matched for age, sex, municipality	In and out patients from all department in four hospitals, no private rheumatologist in the area	No
**Oslo, Norway [19,20]**	1999 - 2009	580 000	≥16	≥ 4 ACR 97 criteria	Yes	General population; 5:1, matched for age, gender and parents ethnicity	1. List of in and out patients from six hospitals. 2. The National Connective Tissue registry. 3.A earlier local cohort. 4. The only two private rheumatologists in the area. 5. Norway's Cause of Death Registry	No
**Oman [21]**	2006 - 2020	~ 4 500 000	All	≥ 4 ACR 97 or SLICC criteria	Yes	Non	Patient from the Royal Hospital and all regional hospitals of the Ministry of Health (MOH), as well as the Sultan Qaboos University Hospital.	No
**Lugo, Spain [22]**	1987 - 2006	NA	≥15	≥ 4 ACR 82 criteria	Yes	Non	In and out patient list of only referral center in the region	No
**Lund, Sweden* [23-26]**	1981 - 2014	~ 200 000	> 15	Clinical diagnosis	Yes	General population; matched age, sex	1. Computerized diagnosis registry at only hospital serving the region 2. Diagnosis registry in primary health care and private practitioners 3. Hospital laboratory (ANA, complement)	Yes
**Taiwan* [27-31]**	1997 - 2011	~ 22 600 000	All	≥ 4 ACR 97 criteria	Yes	General population, age matched	ICD code in The National Health Insurance Research Database that covers more than 96 - 99 % of all residents	No
**Thrace, Turkey [32]**	2003 - 2014	620 447	≥16	≥ 4 ACR 82 criteria	Yes	Non	1. List of in and out patient from one hospital. 2. All state hospital in the region were screened for ICD code. 3. Hospital laboratory (ANA) and pathology department (LN)	No

Study location (ref.)	Inclusion period	Population at risk, n	Age, yrs	Case definition	Chart review	Control group	Sources of Retrieval/Case Ascertainment	Capture recapture
**Georgia, USA [33,34]**	2002 - 2004	NA	All	≥ 4 ACR 82/97 criteria	Yes	General population; life tables; matched age, gender and race	1. Hospitals; rheumatologists, dermatologist - and nephrologists groups in the area. 2. Commercial and hospital-based laboratories (ANA, complement, anti-cardiolipin antibody). 3. Regional pathology laboratories (LN or cutaneous lupus). 5. Lupus research databases. 6. USRDS. 7. Veterans Affairs data. 8. Medicaid claims data. 9. State mortality and hospital discharge data.	Yes
**Michigan, USA [35]**	2002 - 2004	~ 2 400 000	All	≥ 4 ACR 97 criteria	Yes	Non	1. Hospitals/health systems in the area. 2. Private rheumatologists, dermatologists and nephrologists. 3. Commercial laboratories. 4. Medicaid claims. 5. USRDS. 6. Random sample of primary care practices.	Yes
**Minnesota, USA [36]**	1950 - 1992	NA	All	≥ 4 ACR 82 criteria	Yes	General population; life tables	Community diagnostic retrieval system (SLE, Ana, LE cell, false positive syphilis test)	No
**Minnesota , USA [37]**	1993 - 2005	124 277	All	≥ 4 ACR 82 criteria	Yes	General population; life tables; matched age, time of SLE diagnosis, sex	Computerized search in all local providers (Mayo Clinic, Olmsted medical center, local nursing homes and few private practitioner)	No
**Wisconsin, USA [38,39]**	1991 - 2008	77 280	All	≥ 4 ACR 82 criteria	Yes	Non	Community diagnostic retrieval system (SLE); covering all hospital and regional healthcare centers in the region	No

Ref.: References, n: Numbers, ACR: American College of Rheumatology SLE classification criteria, SLICC: The 2012 Systemic Lupus International Collaborating Clinics classification criteria. SLE: Systemic Lupus Erythematosus, ANA: Antinuclear antibody, LE cell: Lupus erythematosus cell test, dsDNA: Anti-double stranded DNA antibody, USRDS: United States Renal Data System, LN: Lupus Nephritis, ICD: International Statistical Classification of Diseases and Related Health Problems, ESRD: End-stage Renal Disease, NA: Not available.
* Patient retrieval at different time periods

References

1. Gonzalez Lucero, L.; Barbaglia, A.L.; Bellomio, V.I.; Bertolaccini, M.C.; Machado Escobar, M.A.; Sueldo, H.R.; Yacuzzi, M.S.; Carrizo, G.A.; Robles, N.; Rengel, S.; et al. Prevalence and incidence of systemic lupus erythematosus in Tucuman, Argentina. *Lupus* **2020**, *29*, 1815-1820, doi:https://dx.doi.org/10.1177/0961203320957719.
2. Flower, C.; Hennis, A.J.; Hambleton, I.R.; Nicholson, G.D.; Liang, M.H.; Barbados National Lupus Registry, G. Systemic lupus erythematosus in an African Caribbean population:

incidence, clinical manifestations, and survival in the Barbados National Lupus Registry. *Arthritis care & research* **2012**, *64*, 1151-1158, doi:https://dx.doi.org/10.1002/acr.21656.
3. Peschken, C.A.; Esdaile, J.M. Systemic lupus erythematosus in North American Indians: A population based study. *Journal of Rheumatology* **2000**, *27*, 1884-1891.
4. Mok, C.C.; Mak, A.; Chu, W.P.; To, C.H.; Wong, S.N. Long-term survival of southern Chinese patients with systemic lupus erythematosus: a prospective study of all age-groups. *Medicine (Baltimore)* **2005**, *84*, 218-224, doi:10.1097/01.md.0000170022.44998.d1.
5. Mok, C.C.; To, C.H.; Ho, L.Y.; Yu, K.L. Incidence and mortality of systemic lupus erythematosus in a southern Chinese population, 2000-2006. *J Rheumatol* **2008**, *35*, 1978-1982.
6. Nossent, J.C. Systemic lupus erythematosus on the Caribbean island of Curaçao: an epidemiological investigation. *Ann Rheum Dis* **1992**, *51*, 1197-1201, doi:10.1136/ard.51.11.1197.
7. Jacobsen, S.; Petersen, J.; Ullman, S.; Junker, P.; Voss, A.; Rasmussen, J.M.; Tarp, U.; Poulsen, L.H.; van Overeem Hansen, G.; Skaarup, B.; et al. A multicentre study of 513 Danish patients with systemic lupus erythematosus. I. Disease manifestations and analyses of clinical subsets. *Clin Rheumatol* **1998**, *17*, 468-477, doi:10.1007/bf01451282.
8. Jacobsen, S.; Petersen, J.; Ullman, S.; Junker, P.; Voss, A.; Rasmussen, J.M.; Tarp, U.; Poulsen, L.H.; van Overeem Hansen, G.; Skaarup, B.; et al. Mortality and causes of death of 513 Danish patients with systemic lupus erythematosus. *Scand J Rheumatol* **1999**, *28*, 75-80, doi:10.1080/030097499442522.
9. Voss, A.; Laustrup, H.; Hjelmborg, J.; Junker, P. Survival in systemic lupus erythematosus, 1995-2010. A prospective study in a Danish community. *Lupus* **2013**, *22*, 1185-1191, doi:10.1177/0961203313498796.
10. Laustrup, H.; Voss, A.; Green, A.; Junker, P. Occurrence of systemic lupus erythematosus in a Danish community: an 8 - year prospective study. *Scandinavian journal of rheumatology* **2009**, *38*, 128-132.
11. Alamanos, Y.; Voulgari, P.V.; Siozos, C.; Katsimpri, P.; Tsintzos, S.; Dimou, G.; Politi, E.N.; Rapti, A.; Laina, G.; Drosos, A.A. Epidemiology of systemic lupus erythematosus in northwest Greece 1982-2001. *Journal of Rheumatology* **2003**, *30*, 731-735.
12. Gergianaki, I.; Fanouriakis, A.; Repa, A.; Tzanakakis, M.; Adamichou, C.; Pompieri, A.; Spirou, G.; Bertsias, A.; Kabouraki, E.; Tzanakis, I.; et al. Epidemiology and burden of systemic lupus erythematosus in a Southern European population: data from the community-based lupus registry of Crete, Greece. *Annals of the Rheumatic Diseases* **2017**, *76*, 1992-2000, doi:https://dx.doi.org/10.1136/annrheumdis-2017-211206.
13. Gudmundsson, S.; Steinsson, K. Systemic lupus erythematosus in Iceland 1975 through 1984. A nationwide epidemiological study in an unselected population. *The Journal of rheumatology* **1990**, *17*, 1162-1167.
14. Ragnarsson, O.; Grondal, G.; Steinsson, K. Risk of malignancy in an unselected cohort of Icelandic patients with systemic lupus erythematosus. *Lupus* **2003**, *12*, 687-691.
15. Iseki, K.; Miyasato, F.; Oura, T.; Uehara, H.; Nishime, K.; Fukiyama, K. An epidemiologic analysis of end-stage lupus nephritis. *American Journal of Kidney Diseases* **1994**, *23*, 547-554.
16. Nossent, H.C. Systemic lupus erythematosus in the Arctic Region of Norway. *Journal of Rheumatology* **2001**, *28*, 539-546.
17. Eilertsen, G.; Becker-Merok, A.; Nossent, J.C. The influence of the 1997 updated classification criteria for systemic lupus erythematosus: epidemiology, disease presentation, and patient management. *J Rheumatol* **2009**, *36*, 552-559, doi:10.3899/jrheum.080574.
18. Eilertsen, G.; Fismen, S.; Hanssen, T.A.; Nossent, J.C. Decreased incidence of lupus nephritis in northern Norway is linked to increased use of antihypertensive and anticoagulant therapy. *Nephrol Dial Transplant* **2011**, *26*, 620-627, doi:10.1093/ndt/gfq435.
19. Lerang, K.; Gilboe, I.M.; Steinar Thelle, D.; Gran, J.T. Mortality and years of potential life loss in systemic lupus erythematosus: a population-based cohort study. *Lupus* **2014**, *23*, 1546-1552, doi:10.1177/0961203314551083.

20. Reppe Moe, S.E.; Molberg, O.; Strom, E.H.; Lerang, K. Assessing the relative impact of lupus nephritis on mortality in a population-based systemic lupus erythematosus cohort. *Lupus* **2019**, *28*, 818-825, doi:https://dx.doi.org/10.1177/0961203319847275.
21. Al-Adhoubi, N.K.; Al-Balushi, F.; Al Salmi, I.; Ali, M.; Al Lawati, T.; Al Lawati, B.S.H.; Abdwani, R.; Al Shamsi, A.; Al Kaabi, J.; Al Mashaani, M.; et al. A multicenter longitudinal study of the prevalence and mortality rate of systemic lupus erythematosus patients in Oman: Oman Lupus Study. *International Journal of Rheumatic Diseases* **2021**, *31*, 31, doi:https://dx.doi.org/10.1111/1756-185X.14130.
22. Alonso, M.D.; Llorca, J.; Martinez-Vazquez, F.; Miranda-Filloy, J.A.; Diaz De Teran, T.; Dierssen, T.; Vazquez-Rodriguez, T.R.; Gomez-Acebo, I.; Blanco, R.; Gonzalez-Gay, M.A. Systemic lupus erythematosus in Northwestern Spain: A 20-year epidemiologic study. *Medicine* **2011**, *90*, 350-358, doi:http://dx.doi.org/10.1097/MD.0b013e31822edf7f.
23. Ingvarsson, R.F.; Landgren, A.J.; Bengtsson, A.A.; Jonsen, A. Good survival rates in systemic lupus erythematosus in southern Sweden, while the mortality rate remains increased compared with the population. *Lupus* **2019**, *28*, 1488-1494, doi:https://dx.doi.org/10.1177/0961203319877947.
24. Jonsson, H.; Nived, O.; Sturfelt, G. Outcome in systemic lupus erythematosus: a prospective study of patients from a defined population. *Medicine* **1989**, *68*, 141-150.
25. Stahl-Hallengren, C.; Jonsen, A.; Nived, O.; Sturfelt, G. Incidence studies of systemic lupus erythematosus in Southern Sweden: increasing age, decreasing frequency of renal manifestations and good prognosis. *Journal of Rheumatology* **2000**, *27*, 685-691.
26. Nived, O.; Bengtsson, A.; Jonsen, A.; Sturfelt, G.; Olsson, H. Malignancies during follow-up in an epidemiologically defined systemic lupus erythematosus inception cohort in southern Sweden. *Lupus* **2001**, *10*, 500-504.
27. Yeh, K.W.; Yu, C.H.; Chan, P.C.; Horng, J.T.; Huang, J.L. Burden of systemic lupus erythematosus in Taiwan: a population-based survey. *Rheumatology International* **2013**, *33*, 1805-1811, doi:https://dx.doi.org/10.1007/s00296-012-2643-6.
28. Chen, Y.-J.; Chang, Y.-T.; Wang, C.-B.; Wu, C.-Y. Malignancy in Systemic Lupus Erythematosus: A Nationwide Cohort Study in Taiwan. *The American Journal of Medicine* **2010**, *123*, 1150.e1151-1150.e1156, doi:10.1016/j.amjmed.2010.08.006.
29. Lin, W.H.; Guo, C.Y.; Wang, W.M.; Yang, D.C.; Kuo, T.H.; Liu, M.F.; Wang, M.C. Incidence of progression from newly diagnosed systemic lupus erythematosus to end stage renal disease and all-cause mortality: a nationwide cohort study in Taiwan. *Int J Rheum Dis* **2013**, *16*, 747-753, doi:10.1111/1756-185x.12208.
30. Yu, K.H.; Kuo, C.F.; Chou, I.J.; Chiou, M.J.; See, L.C. Risk of end-stage renal disease in systemic lupus erythematosus patients: a nationwide population-based study. *International Journal of Rheumatic Diseases* **2016**, *19*, 1175-1182, doi:http://dx.doi.org/10.1111/1756-185X.12828.
31. Lin, C.H.; Hung, P.H.; Hu, H.Y.; Chen, Y.J.; Guo, H.R.; Hung, K.Y. Infection-related hospitalization and risk of end-stage renal disease in patients with systemic lupus erythematosus: A nationwide population-based study. *Nephrology Dialysis Transplantation* **2017**, *32*, 1683-1690, doi:http://dx.doi.org/10.1093/ndt/gfw407.
32. Pamuk, O.N.; Balci, M.A.; Donmez, S.; Tsokos, G.C. The incidence and prevalence of systemic lupus erythematosus in Thrace, 2003-2014: A 12-year epidemiological study. *Lupus* **2016**, *25*, 102-109, doi:10.1177/0961203315603141.
33. Plantinga, L.; Lim, S.S.; Patzer, R.; McClellan, W.; Kramer, M.; Klein, M.; Pastan, S.; Gordon, C.; Helmick, C.; Drenkard, C. Incidence of End-Stage Renal Disease Among Newly Diagnosed Systemic Lupus Erythematosus Patients: The Georgia Lupus Registry. *Arthritis care & research* **2016**, *68*, 357-365, doi:https://dx.doi.org/10.1002/acr.22685.
34. Lim, S.S.; Helmick, C.G.; Bao, G.; Hootman, J.; Bayakly, R.; Gordon, C.; Drenkard, C. Racial Disparities in Mortality Associated with Systemic Lupus Erythematosus - Fulton and DeKalb

Counties, Georgia, 2002-2016. *MMWR - Morbidity & Mortality Weekly Report* **2019**, *68*, 419-422, doi:https://dx.doi.org/10.15585/mmwr.mm6818a4.
35. Somers, E.C.; Marder, W.; Cagnoli, P.; Lewis, E.E.; DeGuire, P.; Gordon, C.; Helmick, C.G.; Wang, L.; Wing, J.J.; Dhar, J.P.; et al. Population-based incidence and prevalence of systemic lupus erythematosus: the Michigan Lupus Epidemiology and Surveillance program. *Arthritis & Rheumatology* **2014**, *66*, 369-378, doi:https://dx.doi.org/10.1002/art.38238.
36. Uramoto, K.M.; Michet, C.J., Jr.; Thumboo, J.; Sunku, J.; O'Fallon, W.M.; Gabriel, S.E. Trends in the incidence and mortality of systemic lupus erythematosus, 1950-1992. *Arthritis & Rheumatism* **1999**, *42*, 46-50.
37. Jarukitsopa, S.; Hoganson, D.D.; Crowson, C.S.; Sokumbi, O.; Davis, M.D.; Michet, C.J., Jr.; Matteson, E.L.; Maradit Kremers, H.; Chowdhary, V.R. Epidemiology of systemic lupus erythematosus and cutaneous lupus erythematosus in a predominantly white population in the United States. *Arthritis Care Res (Hoboken)* **2015**, *67*, 817-828, doi:10.1002/acr.22502.
38. Bartels, C.M.; Buhr, K.A.; Goldberg, J.W.; Bell, C.L.; Visekruna, M.; Nekkanti, S.; Greenlee, R.T. Mortality and cardiovascular burden of systemic lupus erythematosus in a US population-based cohort. *J Rheumatol* **2014**, *41*, 680-687, doi:10.3899/jrheum.130874.
39. Naleway, A.; Davis, M.E.; Greenlee, R.T.; Wilson, D.A.; McCarty, D.J. Epidemiology of systemic lupus erythematosus in rural Wisconsin. *Lupus* **2005**, *14*, 862-866, doi:http://dx.doi.org/10.1191/0961203305lu2182xx.

Supplementary Materials S2

Search strings MEDLINE

1 lupus erythematosus, systemic/ or (systemic lupus or SLE).tw,kf.
2 ((SLICC* or Systemic Lupus International Collaborating Clinics or ACR* or American College of Rhematology or American Rheumatism Association or ARA* or European League Against Rheumatism or EULAR* or lupus or lupusnephritis or SLE) adj4 (criteria or classification*)).tw,kf.
3 ((systemic lupus or SLE) adj2 diagnos*).tw,kf.
4 2 or 3
5 mortality/ or "cause of death"/ or fatal outcome/ or hospital mortality/ or survival rate/ or mortality.fs. or (mortalit* or death* or fatal or fatalit* or surviv*).tw,kf.
6 exp Neoplasms/ or (neoplas* or cancer* or oncol* or malign* or carcinom* or adenocarcinom* or metasta* or leukemia* or myeloma*).tw,kf.
7 5 or 6
8 incidence/ or prevalence/ or (incidence* or prevalence* or population based or ((unselected or un-selected) adj3 cohort*)).tw,kf.
9 (observational study/ or cohort studies/ or follow-up studies/ or longitudinal studies/ or prospective studies/ or retrospective studies/) and (unselected or un-selected).tw,kf.
10 8 or 9
11 1 and 4 and 7 and 10
12 limit 11 to english language
13 Lupus Nephritis/ or (lupusnephritis or ((lupus or lupoid or systemic lupus or SLE) adj2 (nephritis or glomerulonephritis or kidney* or nephropath* or nephro-path*))).tw,kf.
14 exp Renal Replacement Therapy/ or exp Renal Dialysis/ or Kidney Transplantation/ or (dialysis or hemodiafiltration* or hemo-diafiltration* or hemodialysis or ((renal or kidney) adj replacement therap*) or ESRD or end stage renal disease* or end stage kidney disease* or ((kidney or renal) adj2 (transplant* or graft*))).tw,kf.
15 10 and 13 and 14
16 limit 15 to english language
17 12 or 16

Search strings EMBASE

1 systemic lupus erythematosus/ or (systemic lupus or SLE).tw,kw.
2 ((SLICC* or Systemic Lupus International Collaborating Clinics or ACR* or American College of Rhematology or American Rheumatism Association or ARA* or European League Against Rheumatism or EULAR* or lupus or lupusnephritis or SLE) adj4 (criteria or classification*)).tw,kw.
3 ((systemic lupus or SLE) adj2 diagnos*).tw,kw.
4 2 or 3
5 exp mortality/ or exp survival/ or death/ or "cause of death"/ or (mortalit* or death* or fatal or fatalit* or surviv*).tw,kw.
6 exp malignant neoplasm/ or (neoplas* or cancer* or oncol* or malign* or carcinom* or adenocarcinom* or metasta* or leukemia* or myeloma*).tw,kw.
7 5 or 6
8 incidence/ or prevalence/ or population research/ or population based case control study/ or (incidence* or prevalence* or population based or ((unselected or un-selected) adj3 cohort*)).tw,kw.

9 (observational study/ or cohort analysis/ or follow up/ or longitudinal study/ or prospective study/ or retrospective study/) and (unselected or un-selected).tw,kw.

10 8 or 9

11 1 and 4 and 7 and 10

12 limit 11 to conference abstracts

13 11 not 12

14 limit 13 to english language

15 lupus erythematosus nephritis/ or (lupusnephritis or ((lupus or lupoid or systemic lupus or SLE) adj2 (nephritis or glomerulonephritis or kidney* or nephropath* or nephro-path*))).tw,kw.

16 exp renal replacement therapy/ or end stage renal disease/ or kidney graft/ or kidney transplantation/ or (dialysis or hemodiafiltration* or hemo-diafiltration* or hemodialysis or ((renal or kidney) adj replacement therap*) or ESRD or end stage renal disease* or end stage kidney disease* or ((kidney or renal) adj2 (transplant* or graft*))).tw,kw.

17 10 and 15 and 16

18 limit 17 to conference abstracts

19 17 not 18

20 limit 19 to english language

21 14 or 20
